# Botulinum Toxin Type A in the Management of Plantar Fasciitis: A Step Forward in Pain Relief

**DOI:** 10.7759/cureus.90415

**Published:** 2025-08-18

**Authors:** Chaimaa Iziki, Sara Skalli, Lina Lahbabi, Naila Haltout, Samia Karkouri

**Affiliations:** 1 Department of Physical Medicine and Rehabilitation, Ibn Sina University Hospital, Faculty of Medicine and Pharmacy of Rabat, Mohammed V University, Rabat, MAR

**Keywords:** botulinum toxin, corticosteroid injection, heel pain, plantar fasciitis, shockwave therapy

## Abstract

Plantar fasciitis is a prevalent cause of heel pain in adults, particularly among the middle-aged population. It is characterized by microtrauma that exceeds the regenerative capacity of the plantar fascia. Although most cases respond well to conservative treatment, such as stretching exercises, orthotic use, and nonsteroidal anti-inflammatory drugs, some cases progress to chronic stages that require second-line therapies, including extracorporeal shockwave therapy, corticosteroid or platelet-rich plasma injections, or surgical plantar fascia release. This review aims to evaluate the efficacy of botulinum toxin type A (BTA) injections in the management of chronic plantar fasciitis. A narrative review was conducted without any date restrictions, covering the period from 1994 to 2025, through databases including PubMed, Scopus, ScienceDirect, and Google Scholar. Included studies focused on patients with plantar fasciitis unresponsive to conservative treatments, using pain and functional outcomes as endpoints. Three randomized controlled trials demonstrated that BTA injections significantly reduced pain and improved function across short, mid-, and long-term follow-ups. BTA acts at the neuromuscular junctions to reduce muscle tone and modulate pain pathways. BTA injections appear to be a safe and effective option for patients with chronic plantar fasciitis unresponsive to first-line therapies. Image-guided injections (ultrasound or electromyographic) are recommended to enhance accuracy. Further research is necessary to establish standardized protocols regarding the injection site, dosage, guidance technique, and reinjection timing.

## Introduction and background

Plantar fasciitis, also known as plantar fasciopathy, is a frequent cause of heel pain, affecting approximately 4 to 9% of the general population, particularly individuals aged 40 to 60 years old, regardless of their athletic activity [1], and is responsible for approximately one million outpatient visits annually in the United States [2]. The condition stems from repetitive microtears that exceed the reparative capacity of the plantar fascia, ultimately resulting in degenerative changes [3]. Common risk factors include obesity, excessive foot pronation, prolonged standing, and repetitive strain [4].

Anatomically, the plantar fascia is a thick connective tissue band extending from the calcaneal tuberosity to the toes [5]. It plays a crucial role in maintaining the medial longitudinal arch of the foot, absorbing biomechanical stress, and facilitating propulsion during gait. During walking, the fascia stores and releases energy, thereby optimizing movement efficiency [4,5].

Diagnosis is primarily clinical, based on characteristic symptoms such as heel pain that is most pronounced during the first steps in the morning or after periods of rest. Physical examination typically reproduces pain through palpation, passive stretching, or resisted plantar flexion. Imaging is usually not required, but ultrasound may be employed to confirm the diagnosis by assessing fascia thickness, detecting signs of inflammation [6].

Most patients respond favorably to conservative treatments, including stretching, foot orthoses, and non-steroidal anti-inflammatory drugs, with a success rate of 80 to 90% [7]. However, approximately 10% of patients develop chronic, refractory symptoms, requiring more invasive non-surgical approaches, such as corticosteroid injections or platelet-rich plasma (PRP) injections, while a minority may require surgical interventions such as plantar fascia release [3].

Among novel treatment options, botulinum toxin type A (BTA) has shown promising results in musculoskeletal disorders [8]. Produced by Clostridium botulinum, BTA induces temporary chemical denervation by inhibiting acetylcholine release at the neuromuscular junction, thereby reducing mechanical load and allowing functional rest of the plantar fascia [9,10]. Additionally, BTA modulates pain pathways by influencing neuropeptides, such as substance P and CGRP, and by enhancing endogenous opioid mechanisms [11].

This article aims to critically assess the role of BTA injections in alleviating pain and improving function in patients with chronic plantar fasciitis through a comprehensive review of the current literature, highlighting research gaps such as the limited number of high-quality trials, inconsistent protocols, short follow-up durations, and variable outcome measures.

## Review

Materials and methods

A narrative review was performed using the following databases: ScienceDirect, Scopus, PubMed, and Google Scholar. The keywords were: plantar fasciitis, plantar fasciopathy, botulinum toxin, toxin indications, and painful heel. No date restrictions were applied.

Studies were eligible for inclusion if they met the following criteria: Designed as randomized controlled clinical trials, pilot studies, prospective studies, systematic or narrative reviews, meta-analyses, and academic book chapters, written in English or French, and focused on patients diagnosed with plantar fasciitis who were resistant to conventional treatment, with Using validated pain and functional outcome measures.

Exclusion criteria were also clearly defined. Studies were excluded if they consisted solely of conference abstracts, posters, or oral presentations, as these formats often lack peer review and detailed methodology. Additionally, studies that focused on other causes of heel pain or those lacking objective outcome measures were excluded to preserve the specificity and scientific rigor of the narrative review.

The selection process aimed to capture both clinical and methodological diversity in order to better evaluate the efficacy and therapeutic potential of BTA in plantar fasciitis.

Results

Although the literature on the use of BTA injections in plantar fasciitis remains limited, three key studies, primarily randomized controlled trials, stand out for their methodological quality and relevance.

Huang et al. (2010) conducted a randomized, double-blind controlled trial to evaluate the efficacy of a local BTA injection into the plantar fascia. Participants in the intervention group received 50 IU of BTA, while those in the control group received a saline injection, with both procedures performed under ultrasound guidance. In the short and medium term, the BTA group showed a statistically significant improvement in pain, with the Visual Analog Scale (VAS) score decreasing from 5.4 to 3.4 (p < 0.001). Additionally, ultrasound measurements revealed a reduction in plantar fascia thickness (from 5.5 mm to 4.2 mm and 3.6 mm) (p < 0.001). Long-term outcomes, however, were not assessed [12].

Elizondo-Rodriguez et al. (2013) conducted a randomized controlled trial comparing intramuscular BTA injections into the triceps surae with peri-fascial corticosteroid injections. Nineteen patients received 50 IU BTA injections in the soleus and 100 IU in each gastrocnemius, while 17 control participants were treated with a peri-fascial injection of 8 mg dexamethasone combined with 2 ml of 2% lidocaine, administered using anatomical landmarks. Both groups followed a stretching program. Patients were assessed at 15 days, at one, two, four, and six months. VAS pain scores in the BTA group improved significantly from 7.1 to 1.9 (p=0.0004), with sustained benefits at medium-term follow-up (p=0.0005). However, there were no significant differences between the two groups regarding functional outcome as measured by the Maryland Foot Score [13].

Ahmad et al. (2016) conducted a randomized, double-blind controlled trial assessing the effect of 100 IU of BTA injected under electromyographic guidance into the flexor digitorum brevis muscle and at the most painful area of the plantar fascia. The control group received an equivalent volume of saline injection under the same conditions. All participants underwent supervised rehabilitation sessions. At six and 12 months, the BTA group showed significant improvement in both function and pain: the Foot and Ankle Ability Measure (FAAM) score increased from 36.3 to 73.8/100 (p < 0.05), while VAS scores decreased from 7.2 to 3.6 at mid-term and 2.9 at long-term follow-up (p = 0.01) [14].

Various results of these studies are summarized in Table 1 and Table 2.

**Table 1:** Comparative description of botulinum toxin injection techniques, key outcome measures, and follow-up periods in the included studies BTA: Botulinum Toxin Type A; VAS: Visual Analogue Scale; MFS: Maryland Foot Score; FAAM: Foot and Ankle Ability Measure

Author (Year)	Groups	Study Design	Intervention/Dose/Injection Site	Outcome Measures	Methodological Details
Huang (2010) [12]	25 patients in the BTA group and 25 patients in the placebo group	Randomized double-blind controlled trial	BOTOX®: 50 U/ml 50 U injected into plantar fascia; placebo: 1 ml saline solution; Ultrasound-guided	VAS pain score/plantar fascia thickness measured by ultrasound (including subcutaneous fat)	Evaluation at three weeks and three months
Elizondo-Rodríguez (2013) [13]	40 participants – 4 lost to follow-up Group A: 19 BTA Group B: 17 corticosteroid control	Randomized double-blind controlled trial, single center	BTA: 100 U in each gastrocnemius and 50 U in soleus Control: 8 mg dexamethasone + 2 ml 2% lidocaine injected into the medial surface of plantar fascia; injection guided by anatomical landmarks	VAS (visual analog scale) Color scale - red-green MFS	Evaluations performed by a blinded investigator at 15 days, one month, three months, and six months; associated care: Patients received stretching advice/education starting day 7 post-injection
Ahmad (2016) [14]	25 patients in the BTA group; 25 patients in the placebo group	Randomized double-blind controlled monocentric trial	XEOMIN® 100 U in the flexor digitorum brevis muscle, adjacent to the painful point on the distal medial part of the calcaneus; placebo: 1 ml Control by EMG stimulation; dilution not specified	VAS at six and 12 months; FAAM at six and 12 months (two different authors compared the results	Trained operator; rehabilitation for a minimum of six weeks: stretching, cryotherapy, ultrasound, massage extended another six weeks if needed

**Table 2:** Summary of clinical outcomes of BTA injections for chronic plantar fasciitis EMG: Electromyography; VAS: Visual Analogue Scale; BTA: Botulinum Toxin Type A

Author (Year)	Dose & Injection Site	Pain Measure (VAS)	Baseline	Short Term (1-3 Months)	Medium Term (Six Months or More)	Function Measure	Baseline	Post-treatment (Short/Medium Term)
Huang (2010) [12]	50 U into plantar fascia (ultrasound-guided)	VAS	5.4	3.4 (3 weeks)	Not assessed	Plantar fascia thickness (mm)	5.5 mm	4.2 mm (3 weeks), 3.6 mm (3 months)
Elizondo-Rodríguez (2013) [13]	100 U gastrocnemius + 50 U soleus (anatomical landmarks)	VAS	7.1	1.9 (15 days)	1.9 (6 months)	Maryland Foot Score	Not reported	No significant difference vs. control
Ahmad (2016) [14]	100 U flexor digitorum brevis (EMG-guided)	VAS	7.2	3.6 (6 months)	2.9 (12 months)	Foot and Ankle Ability Measure	36.3 / 100	73.8 / 100 (6-12 months)

A 2016 meta-analysis encompassing 22 randomized controlled trials further supported the analgesic effect of BTA. The review highlighted significant pain relief within the first six months and functional improvement within the first two months post injection. Importantly, no major adverse events were reported, reinforcing the safety of the intervention [15].

Discussion

The management of plantar fasciitis relies on conservative non-invasive approaches aimed at addressing biomechanical overload and modifiable risk factors. Standard first-line therapies include rest, reduction of prolonged standing, and adapting physical activity [16], ice massages, nonsteroidal anti-inflammatory drugs, plantar fascia-specific stretching exercises, strengthening of the intrinsic foot muscles, and the use of custom shock-absorbing or corrective orthotic devices [17].

For patients with chronic symptoms unresponsive to these interventions, second-line treatments such as corticosteroid injections, PRP injections [18], prolotherapy (dextrose-based injections), and dry needling [19]. Extracorporeal shock wave therapy or even surgical options like plantar fascia release or gastrocnemius recession may be considered [20].

Compared to other injectable treatments used for plantar fasciitis, such as corticosteroids or PRP, BTA offers several notable advantages. It often provides longer-lasting relief [21], without the common side effects associated with corticosteroids (skin atrophy, tendon rupture) [22]. Unlike PRP, BTA does not require blood sampling or specific preparation, making it easier to use and potentially more accessible, particularly in facilities without a laboratory or centrifugation equipment [23]. 

While BTA has shown promising efficacy in chronic plantar fasciitis, its safety profile should also be considered. Adverse effects are generally rare and mild, typically including transient local pain, bruising, temporary muscle weakness, or mild allergic reactions [24,25].

BTA has emerged as a promising therapeutic alternative in this context. The reviewed studies consistently reported improvements in both pain and function following BTA injections.

These effects are likely attributable to two complementary mechanisms: a reduction in mechanical stress via transient chemodenervation of muscles contributing to fascial tension and a direct modulation of pain pathways. BTA has been shown to inhibit the release of pain-related neuropeptides such as substance P and CGRP, while also enhancing endogenous opioid activity [9-11].

Despite these promising findings, several limitations should be noted. The protocols used across the studies varied widely in terms of BTA formulation, dosage, injection sites, and guidance techniques (ultrasound providing direct anatomical visualization vs electromyography that relies on active motor point detection). Such heterogeneity hampers the ability to draw definitive conclusions or establish standardized treatment guidelines. Moreover, most studies included small sample sizes and short-to-medium follow-up durations.

Nonetheless, the overall methodological quality of the included trials is high, and the results are consistent with data from other musculoskeletal conditions where BTA has shown benefit, particularly in lateral epicondylitis, which shares a similar pathophysiological mechanism to plantar fasciitis. In that context, BTA facilitates enthesis rest, promotes healing, and relieves pain [26]. Additional studies have also demonstrated its utility in managing myofascial pain syndrome, piriformis syndrome, osteoarthritis, and chronic spinal and joint pain [9,27].

Taken together, while current findings are promising, further high-quality studies with larger sample sizes and standardized protocols are needed to fully substantiate the safety and effectiveness of BTA in chronic plantar fasciitis refractory to conventional treatments.

## Conclusions

BTA appears to be a promising therapeutic option for the treatment of chronic plantar fasciitis unresponsive to conventional conservative management. Its dual mechanism, reducing mechanical strain and modulating nociceptive pathways, offers both structural and symptomatic relief. The use of ultrasound or electromyographic guidance enhances injection accuracy and may optimize clinical outcomes. While short and medium-term results are encouraging, further high-quality studies are warranted to establish standardized protocols regarding the dosage, injection site, guidance technique, and the need for or frequency of reinjection intervals. In addition, future research should evaluate the long-term safety profile of BTA and directly compare its efficacy and cost-effectiveness with other established treatments, such as corticosteroids and PRP.

## References

[1] Taunton JE, Ryan MB, Clement DB, McKenzie DC, Lloyd-Smith DR, Zumbo BD (2002). A retrospective case-control analysis of 2002 running injuries. Br J Sports Med.

[2] Fraser JJ, Glaviano NR, Hertel J (2017). Utilization of physical therapy intervention among patients with plantar fasciitis in the United States. J Orthop Sports Phys Ther.

[3] Young CC, Rutherford DS, Niedfeldt MW (2001). Treatment of plantar fasciitis. Am Fam Physician.

[4] Stiglitz Y (2014). Plantar aponeurositis and retraction of the achilles-plantar system (Article in French). Entretiens de Bichat.

[5] Cutts S, Obi N, Pasapula C, Chan W (2012). Plantar fasciitis. Ann R Coll Surg Engl.

[6] Mohseni-Bandpei MA, Nakhaee M, Mousavi ME, Shakourirad A, Safari MR, Vahab Kashani R (2014). Application of ultrasound in the assessment of plantar fascia in patients with plantar fasciitis: a systematic review. Ultrasound Med Biol.

[7] Davis PF, Severud E, Baxter DE (1994). Painful heel syndrome: results of nonoperative treatment. Foot Ankle Int.

[8] Singh JA (2013). Use of botulinum toxin in musculoskeletal pain. F1000Res.

[9] Khenioui H, Houvenagel É, Catanzariti JF, Guyot MA, Agnani O, Donze C (2016). Interest in intra-articular injections of botulinum toxin (Article in French). Revue du Rhumatisme.

[10] De Sèze MP, De Sèze M, Dehail P, Joseph PA, Lavignolle B, Barat M, Mazaux JM (2003). Botulinum toxin and musculoskeletal pain (Article in French). Annales de Réadaptation et de Médecine Physique.

[11] Poulain B, Lonchamp E, Jover E, Popoff MR, Molgó J (2009). Mechanismsof action of botulinum toxins and neurotoxins. Annales de Dermatologie et de Vénéréologie.

[12] Huang YC, Wei SH, Wang HK, Lieu FK (2010). Ultrasonographic guided botulinum toxin type A treatment for plantar fasciitis: an outcome-based investigation for treating pain and gait changes. J Rehabil Med.

[13] Elizondo-Rodriguez J, Araujo-Lopez Y, Moreno-Gonzalez JA, Cardenas-Estrada E, Mendoza-Lemus O, Acosta-Olivo C (2013). A comparison of botulinum toxin a and intralesional steroids for the treatment of plantar fasciitis: a randomized, double-blinded study. Foot Ankle Int.

[14] Ahmad J, Ahmad SH, Jones K (2016). Treatment of plantar fasciitis with botulinum toxin. Foot Ankle Int.

[15] Tsikopoulos K, Vasiliadis HS, Mavridis D (2016). Injection therapies for plantar fasciopathy ('plantar fasciitis'): a systematic review and network meta-analysis of 22 randomised controlled trials. Br J Sports Med.

[16] Fulpius T (2014). Talalgia (plantar fasciitis): how to manage it? (Article in French). Revue Médicale Suisse.

[17] Aloulou I, Dziri C, Kharrat O, Lebib S, Miri I, Ben Salah FZ, Elleuch M (2013). What are the different medical therapeutic modalities for plantar fasciitis. Med Chir Pied.

[18] Herber A, Covarrubias O, Daher M, Tung WS, Gianakos AL (2024). Platelet rich plasma therapy versus other modalities for treatment of plantar fasciitis: a systematic review and meta-analysis. Foot Ankle Surg.

[19] Geoffroy T (2025). Foot and ankle pathologies in tennis players (Article in French). Revue du Podologue Volume 21, Issue.

[20] Goff JD (2011). Diagnosis and treatment of plantar fasciitis. Am Fam Physician.

[21] Díaz-Llopis IV, Rodríguez-Ruíz CM, Mulet-Perry S, Mondéjar-Gómez FJ, Climent-Barberá JM, Cholbi-Llobel F (2012). Randomized controlled study of the efficacy of the injection of botulinum toxin type A versus corticosteroids in chronic plantar fasciitis: results at one and six months. Clin Rehabil.

[22] Latt LD, Jaffe DE, Tang Y, Taljanovic MS (2020). Evaluation and treatment of chronic plantar fasciitis. Foot Ankle Orthop.

[23] Dashore S, Chouhan K, Nanda S, Sharma A (2021). Preparation of platelet-rich plasma: national IADVL PRP taskforce recommendations. Indian Dermatol Online J.

[24] Naumann M, Jankovic J (2004). Safety of botulinum toxin type A: a systematic review and meta-analysis. Curr Med Res Opin.

[25] Dressler D, Adib Saberi F (2005). Botulinum toxin: mechanisms of action. Eur Neurol.

[26] Morré H, Keizer S, Van Os J (1997). Treatment of chronic tennis elbow with botulinum toxin. Lancet.

[27] Moore C, Hulsopple C, Boyce B (2020). Utilization of Botulinum toxin for musculoskeletal disorders. Curr Sports Med Rep.

